# The effect of white noise on sleep quality and fatigue in community-dwelling older adults: a randomized controlled trial

**DOI:** 10.1186/s12877-026-07311-2

**Published:** 2026-05-02

**Authors:** Seyed Amirhossein Vahhabzadeh Mousavi, Maryam Salehian, Seyed Reza Mazlom

**Affiliations:** 1 MSc in Geriatrics Nursing, School of Nursing and Midwifery, Mashhad University of Medical Sciences, Mashhad, Iran; 2 Department of Geriatric Nursing, Nursing Care Research Center, Faculty of Nursing and Midwifery, Mashhad University of Medical Sciences, Mashhad, Iran; 3 Department of Medical Surgical Nursing, School of Nursing and Midwifery, Mashhad University of Medical Sciences, Mashhad, Iran; 4 Nursing and Midwifery School, Dr. Kharazmi Educational Building, University Campus, East Gate of Ferdowsi University of Mashhad, Azadi Square, Mashhad, Khorasan Razavi Iran

**Keywords:** White noise, Sleep quality, Fatigue, older adults, Alternative medicine

## Abstract

**Background:**

With increasing global life expectancy, population aging has become a major public health issue. Poor sleep quality and fatigue are prevalent among older adults, negatively impacting their quality of life and daily functions. While pharmacological interventions for sleep disorders are common, they carry significant side effects, especially in the older individuals. Non-pharmacological alternatives like white noise are simple, safe, and cost-effective, yet evidence for their effectiveness among community-dwelling older adults remains limited. This study aims to evaluate the effectiveness of white noise on improving sleep quality and reducing fatigue among community-dwelling older adults.

**Methods:**

This parallel RCT was conducted among 60 individuals 65 years and older attending healthcare centers in Mashhad, Iran. After the initial screening, eligible participants were randomly assigned into two groups. Both groups received standard sleep hygiene recommendations based on provincial guidelines. Additionally, the intervention group was provided with audio options and were instructions to use them over the next 30 consecutive nights. Constant follow-ups ensured adherence. Sleep quality and fatigue were measured using PSQI and IFS pre- and post-intervention. Group differences over time were examined using repeated measures and baseline adjusted analyses. Descriptive and inferential data analysis was performed using SPSS 27 at a significance level of *p* < 0.05.

**Results:**

Post-intervention results show that there is a statistically significant difference between the two groups in both PSQI and IFS scores. Our results show significant improvement in the intervention group’s PSQI (from 11.5 ± 2.8 to 9.2 ± 3.1, *P* < 0.001) and IFS scores (from 35.1 ± 3.5 to 32.4 ± 4.9, *P* < 0.001), with no significant changes in the control group.

**Conclusions:**

Our findings suggest that the use of white noise can result in improving sleep quality and may be helpful in reducing fatigue in community-dwelling older adults and can be recommended as an low effort, low-cost and safe strategy to enhance sleep and reduce fatigue in older individuals.

**Trial registration:**

(IRCT20240812062732N1), Date of registration August 28, 2024.

**Supplementary Information:**

The online version contains supplementary material available at 10.1186/s12877-026-07311-2.

## Introduction

With the recent scientific advancements, better quality of life and easier access to healthcare services, the number of older adults has grown significantly. According to WHO, the number of people aged 60 and above is expected to double by 2050, reaching 2.1 billion [1]. In Iran, by 2036, the population of people 65 years and older will reach 13.5 million, constituting 14.4% of the total population [2, 3].

With normal aging, prevalence of physical and psychological complications will increase, among which sleep disturbances and fatigue are particularly prominent [4, 5]. According to our research, 43.6% of older adults suffer from poor sleep quality (PSQI ≥ 6), and it appears to become more common with advancing age, with prevalence around 48% in populations aged ≥ 70 years [4]. In addition, around 50% of people aged 70 and 75% of people aged 85 have reported increased fatigue [6]. These issues not only compromise the quality of life in older adults but also contribute to the progression of frailty and dependency in this population [4, 7, 8].

Examples of sleep problems in older adults include but are not limited to, difficulty falling asleep, frequent awakenings and reduced restorative sleep, which, based on our findings, can often be accompanied by persistent fatigue [6, 7, 9, 10]. Sleep disturbances are among the most common and modifiable contributors to fatigue in older adults [10]. Fatigue can be described as a multidimensional phenomenon that affects cognitive, emotional, and physical functioning [6, 11, 12].

Given evidence from recent observational and interventional studies indicating that improvements in sleep quality are associated with reductions in perceived fatigue, we decided to examine fatigue alongside sleep quality to allow for a more comprehensive assessment of the clinical relevance of sleep-focused interventions and to better reflect outcomes that matter to older adults’ daily lives.

Although the common ways to manage sleep problems in older adults usually involve pharmacological interventions, they often come with high risk of adverse effects [13, 14]. It also needs to be mentioned that a significant number of older adults are currently taking multiple medications and are more prone to added risk of medication interactions in comparison to other age groups [15, 16].

As a result, there has been a growing interest in Complementary and Alternative Medicine and non-pharmacological approaches to improve sleep and reduce fatigue in this population. Some of these approaches include sleep hygiene education, regular physical activity, cognitive behavioral therapy (CBT), relaxation-based techniques such as auditory stimulation techniques, hypnosis, accoucheur, acupressure, yoga, and many others [17, 18]. While these approaches can be effective, they often require sustained behavioral changes, cognitive engagement, access to trained professionals and some amount of physical mobility which may limit their feasibility for some older adults. Among these, auditory stimulation techniques like white noise have gained attention for their potential to mask environmental disturbances and promote sleep continuity [19–21].

White noise is a consistent sound containing all audible frequencies at equal intensity, which have been shown to aid in reducing sleep onset latency and improving sleep quality [20, 22, 23].

Some of the key advantages of white noise interventions include their passiveness and low cognitive demand, good tolerability among older individuals, very low effort to be used, low cost and easily scalable. Additionally, they are safe for polypharmacy-prone populations and can be effective for masking environmental noise without behavioral burden [19, 24, 25]. Reviewing previous studies has shown that the use of white noise can be beneficial in lowering the activity of sympathetic nervous system and thus reducing blood pressure and heart rate [26]. Previous studies have also shown positive effects of white noise on sleep among various populations, including those in critical care units and post-operative settings [22, 26–28], yet evidence regarding its efficacy in community-dwelling older adults remains limited and inconclusive.

Considering the worldwide impact of sleep-related problems and increased fatigue among the growing older adult population and their quality of life, along with the potential of white noise as a safe, low-cost, and non-invasive intervention, further investigation on this subject seems necessary. To address this gap, the present randomized controlled trial (RCT) was conducted to examine the effect of white noise on sleep quality and fatigue among community dwelling older individuals visiting the healthcare centers near their home.

### Goals & objectives

The Primary Objectives of this study are: (1) determine the effect of using white noise on sleep quality in older adults; (2) determine the effects of improving sleep quality on fatigue in older adults.

Secondary Objectives of this study are: (1) determine the effect of using white noise on sleep latency; (2) determine the effect of using white noise on total sleep duration of older adults.

## Materials and methods

This parallel randomized controlled trial was conducted among individuals 65 years and older, visiting healthcare centers. Participants meeting the inclusion criteria were randomly assigned to intervention and control groups. The control group received routine sleep hygiene education according to health center guidelines. In addition to routine care, the intervention group used white noise as they were instructed. After trial commencement, with the permission of the ethics committee and the approval of IRCT we decided to replace the Mini-Mental State Examination questionnaire with Abbreviated Mental Test Score (AMTS) due to it being more suitable for our study population.

### Participants and recruitment

Participants were recruited through convenience sampling from six urban healthcare centers, covered by the main Health Center No. 1 (out of 5) in Mashhad, Iran. Study recruitment was conducted from March to June 2025. Older adults who visited the selected centers were screened for eligibility based on predefined inclusion and exclusion criteria. Eligibility included individuals 65 years or older, willing to participate, had an active health record at the visited health center, and had signs of sleep disturbance (PSQI ≥ 6) and fatigue (IFS ≥ 30). Exclusion criteria included not being oriented based on AMTS score (score < 7), a documented history of cancer, major cardiac disease, depression, chronic pulmonary disease, hypothyroidism, multiple sclerosis, Parkinson’s disease, rheumatoid arthritis, or Guillain–Barré syndrome; withdrawal of consent or unwillingness to continue participation in the study; non-adherence to the intervention protocol, defined as missing more than three consecutive sessions of white noise exposure; significant changes in physical or cognitive status that could interfere with participation or outcome measurement; and inability to pass the whisper test which was conducted on all participants before enrolment.

### Participant flow and randomization

**Fig. 1:**
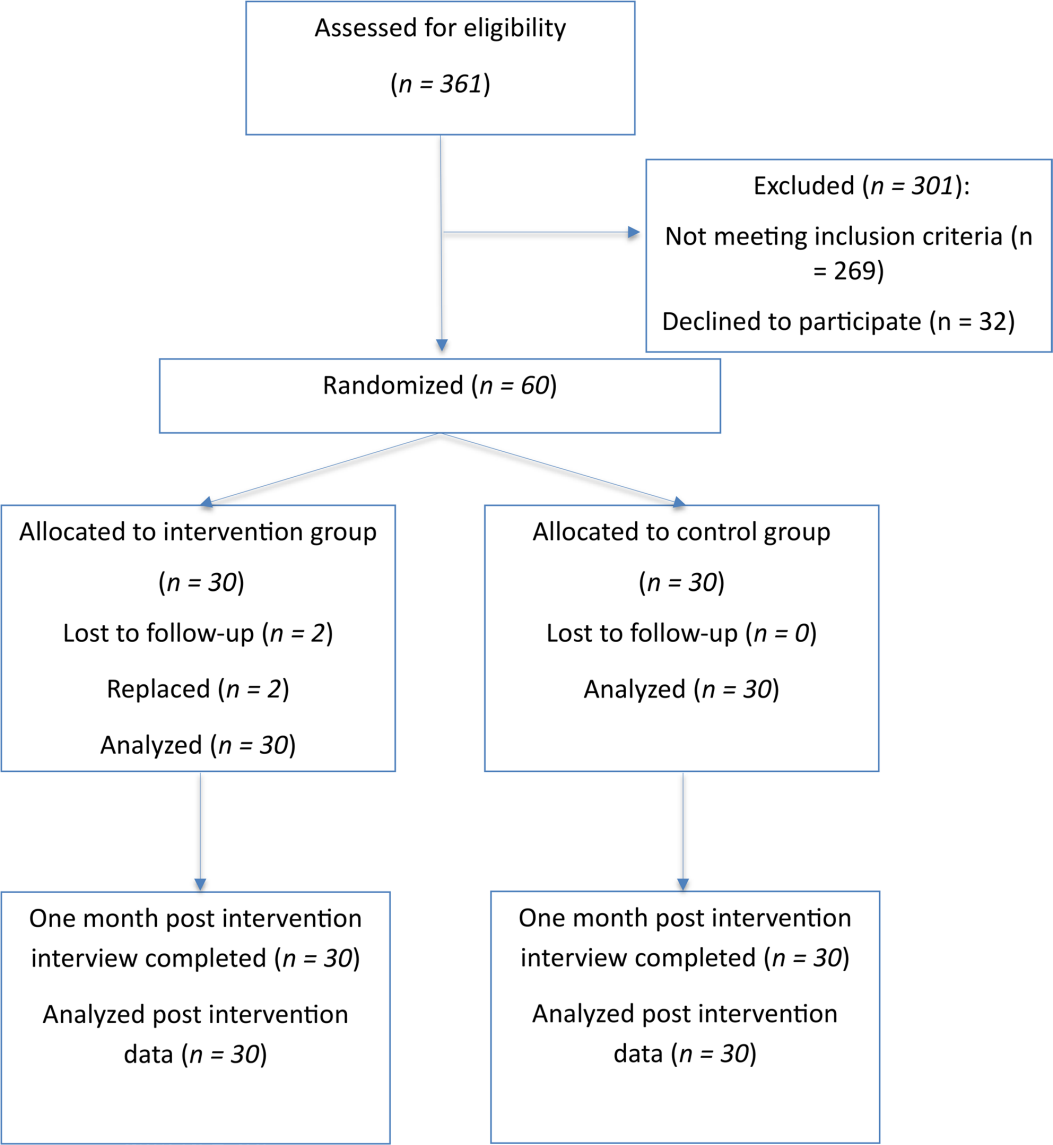
Study flow diagram

A total of 361 older adults were screened for eligibility as shown in Fig. 1. 269 out of 361 did not meet the inclusion criteria and 32 declined to participate in our study. 60 individuals who met the inclusion and exclusion criteria were randomly assigned into two groups with a 1:1 ratio. In the early phase of the study, two participants from the intervention group, withdrew from the study before completing the intervention due to personal reasons. At that time, participant recruitment was still ongoing; to maintain group balance, two additional eligible participants were enrolled and randomized to the intervention group. No additional dropouts occurred in the control group. All participants who completed the study were included in the final analysis. Randomization was performed using a permuted block method with a block size of six. The randomization sequence was prepared in advance and was generated via the online tool SealedEnvelope.com. Group assignment was concealed using sealed, opaque, sequentially numbered envelopes. After initial screening, each eligible participant was assigned into one of the two groups based on the card drawn from the envelopes, ensuring allocation concealment and a 1:1 allocation ratio. Randomization and allocation were conducted independently from the intervention and outcome assessment teams to minimize bias.

### Intervention training and follow-up

The protocol of this RCT was developed based on previous studies such as Genç et al. (2020), Oshvandi et al. (2020) and Farokhnezhad et al. (2016). Additionally, to ensure the safety of our participants, the sound intensity and duration were maintained at a level commonly considered safe by WHO for prolonged exposure and consistent with environmental noise recommendations for sleep settings [29]. With all of these considerations, our intervention was designed to be low-cost, non-invasive and easily accessible, making it feasible for routine use in community and home care settings. Participants in the intervention group were instructed by registered nurses to listen to their own preferred white noise from the provided four noises, using their own mobile phone speakers for 30 min each night immediately after getting in bed. This listening duration was selected based on prior evidence suggesting that longer exposure times do not provide any additional benefits and may cause discomfort [20]. Intervention duration was set to 30 consecutive nights to align with the one-month recall period of the PSQI assessment. The white noise files were preloaded onto participants’ phones and standardized across all participants to ensure consistency. Following the safety suggestions in similar studies and to ensure that all participants listen to the provided sounds, approximately at 40-50dB, all participants were instructed to set the volume level at 50–60% and to set their phone at 1 m distance from their heads. This setup was based on commonly used smartphone models in the study population and prior auditory research regarding speaker output and sound travel. According to a previous study, this volume range is considered optimal because it is loud enough that can help mask environmental peak noises but quiet enough to avoid causing arousal or hearing fatigue [23]. The intervention was implemented in the participants’ own living environments and integrated into their natural bedtime routine to enhance ecological validity and adherence. Background environmental noise was not controlled in order to reflect real-world sleeping conditions. Participants were monitored periodically to ensure compliance. Nightly reminder SMS texts, listening logs, weekly telephone follow-ups and participant reports were used to track adherence. If participants missed more than three consecutive sessions, they were withdrawn from the study as per the exclusion criteria. Both groups received usual care, provided by healthcare providers at the healthcare centers, consisting of standard health education and sleep hygiene recommendations based on national guidelines in the form of educational pamphlets. No additional interventions were introduced to the control group during the study period.

### Outcomes measurement tools and questionnaires

Per our exclusion criteria, we have used the Abbreviated Mental Test Score (AMTS) questionnaire to assess our participants’ ability to give informed consent and to assess their ability to carry out the intervention by themselves. This tool was developed by Dr. Hodkinson in 1972 and was translated and validated into Persian by Bakhtiari et al. in 2014 [30, 31]. A global score between 0 and 10 is provided by the AMTS; a higher score denotes fewer mental complications. A global AMTS score ≥ 7 was used to identify mentally alert individuals. The Pittsburgh Sleep Quality Index (PSQI), a widely validated tool for assessing subjective sleep quality over the course of the previous month, was used to measure sleep quality as the study’s first primary outcome. A global score between 0 and 21 is provided by the PSQI; a higher score denotes poorer sleep quality. A global PSQI score ≥ 6 was used to identify poor sleepers. This tool was developed by Buysse et al. in 1989 and was translated and validated into Persian by Kakouei et al. in 2010 [32, 33]. The second primary outcome was fatigue, measured using the Iowa Fatigue Scale (IFS), which evaluates fatigue intensity, functional impact, and energy levels in daily life. The global IFS score ranges from 11 to 55, with higher scores indicating higher fatigue levels. A global IFS score ≥ 30 was used to identify fatigued individuals. This tool was developed by Dr. Hartz et al. in 2003 and was translated and validated into Persian by Kasnavi in 2014 [34, 35]. Secondary outcomes were measured as part of the PSQI standard questionnaire. Both primary and secondary outcomes were measured at baseline and at the end of the 30-day intervention period in both groups. These tools are validated and commonly used in older adult populations. All questionnaire used in this study have previously demonstrated strong psychometric properties in older populations and have been translated and culturally adapted for use in Persian-speaking populations. The reliability (Internal Consistency) of the PSQI and IFS questionnaires in our study was assessed using Cronbach’s alpha. The Cronbach’s α values were 0.81 for the PSQI and 0.74 for the IFS. Permission to use the mentioned tools has been obtained from their rightful owners. No formal minimal important difference (MID) thresholds were available for the PSQI or IFS; therefore, statistical significance and effect sizes were used to interpret the impact of the intervention.

### Blinding and sample size

Due to the nature of the intervention, blinding of participants was not feasible. However, to minimize bias, outcome data were coded, and the data analysis was conducted by a researcher who was blinded to group assignments. Before the sampling phase, the sample size was calculated using the formula for comparing two independent means based on our two primary outcomes (sleep quality and fatigue) from prior white-noise sleep intervention studies. The primary effect size estimate was derived from the study by Genç et al. (2020), assuming a 95% confidence level and 80% statistical power, which resulted in a minimum required sample size of 14 (based on sleep quality) and 23 (based on fatigue) participants per group. To account for a potential dropout rate of 30% due to the strict exclusion criteria, the final sample size was set to 30 participants per group. Additionally, after the study was conducted, we used G*Power version 3.1 (Heinrich-Heine-University, Düsseldorf, Germany) with confidence level of 95% and a statistical power of 90% to ensure more accurate results. Minimum required sample size in our second calculation was 19 (based on sleep quality) and 30 (based on fatigue) participants per group [28, 36–39].

### Statistical analysis

The reporting of this trial follows the CONSORT guideline. To compare demographic and baseline characteristics, descriptive statistics were used. The normality of continuous variables was assessed using the Shapiro–Wilk and Kolmogorov–Smirnov tests. For between-group comparisons, independent samples t-tests or Mann–Whitney U tests were used, and for continuous variables, Chi-square and Fisher’s exact tests were applied for categorical variables. When necessary, Wilcoxon signed-rank or paired t-tests were used to examine within-group changes. Repeated measures analyses were performed as well. Post-intervention scores were compared between the two groups using analysis of covariance (ANCOVA) models. To evaluate whether changes in outcomes over time differed between groups, time × group interaction effects were examined using repeated-measures analysis and ANCOVA models adjusted for baseline values and potential confounding factors. Significance was set at *p* < 0.05 (two-tailed). SPSS version 27.0 was used to conduct statistical analysis (IBM Corp., Armonk, NY, USA).

### Ethics approval and consent to participate

Our study was conducted in accordance with the ethical standards of the Declaration of Helsinki (2013 revision).Prior to the sampling phase, institutional ethics approval was obtained from the Ethics Committee of Mashhad University of Medical Sciences. The study protocol was submitted and approved by Iranian Registry of Clinical Trials. Additionally, prior to enrollment, written informed consent was obtained from all participants. Participants were assured of the confidentiality of their information, their right to withdraw from the study at any time without penalty, and that participation in this study was entirely voluntary.

## Results

### Baseline demographic characteristics

Table 1 provides information related to the demographic characteristics and baseline measurements of participants. At baseline, both groups had similar demographic characteristics including medication use. The mean age (± SD) in the intervention group was 69.6 ± 5.6 years and 71.0 ± 5.3 years in the control group (Mann–Whitney U = 351.0, z = -1.473, *P* = 0.141). The majority of participants in both groups were female (intervention: 70% and control: 73.3%; χ² (1) = 0.082, *P* = 0.774), married (73.3% and 70.0% respectively; χ² (2) = 0.090, *P* = 1.000), and unemployed (90.0% and 83.3% respectively; χ² (2) = 4.609, *P* = 0.100). In terms of educational, 20% of participants in the intervention group and 13.3% in the control group had a university degree (Mann–Whitney U = 393.0, z = -0.864, *P* = 0.388). Additionally, comparison of the AMTS scores shows that both groups had similar cognitive functions (intervention: 9.1 ± 1.06 and control: 8.7 ± 1.2; Mann–Whitney U = 376.0, z = -1.166, *P* = 0.244). No statistically significant differences between the two groups were observed in any of the baseline characteristics (*P* > 0.05).

**Table 1:** Baseline Characteristics of Older Adults

Characteristic	Intervention Group (*n* = 30)	Control Group (*n* = 30)	Statistic	*P* value
AMTS score ^a^	**mean ± (SD)** 9.1 ± (1.06)	**mean ± (SD)** 8.7 ± (1.2)	U = 376.0 ( z = -1.166)	0.244
Gender (Female)	**n (%)** 21 (70.0%)	**n (%)** 22 (73.3%)	χ² (1) = 0.082	0.774
Age in years -65–69.9 -70–74.9 -75+	**n (%)** 19 (63.4%) 6 (20.0%) 5 (16.6%)	**n (%)** 10 (33.4%) 13 (43.3%) 7 (23.3%)	U = 351.0 ( z = -1.473)	0.141
Marital Status -Married, living together Never -Not married, Widowed, Divorced, Separated	**n (%)** 22 (73.3%) 8 (16.7%)	**n (%)** 21 (70.0%) 9 (30.0%)	χ² (2) = 0.090	1.000
Education Level -Illiterate -Attended school, high school diploma -University degree	**n (%)** 4 (13.3%) 20 (66.7%) 6 (20.0%)	**n (%)** 3 (10.0%) 23 (76.7%) 4 (13.3%)	U = 393.0 ( z = -0.864)	0.388
Employment Status -Home maker -Retierd -Working	**n (%)** 12 (40.0%) 15 (50.0%) 3 (10.0%)	**n (%)** 18 (60.0%) 7 (23.3%) 5 (16.7%)	χ² (2) = 4.609	0.100
Type of home -Apartment - Villa	**n (%)** 27 (90.0%) 3 (10.0%)	**n (%)** 26 (80.0%) 4 (20.0%)	χ² (1) = 1.176	0.278
Living with -Alone -With spouse -With children -More than two people	**n (%)** 5 (16.7%) 17 (56.7%) 4 (13.3%) 4 (13.3%)	**n (%)** 6 (20.0%) 15 (50.0%) 4 (13.3%) 5 (16.7%)	χ² (3) = 0.327	0.959
Underlying chronic condition(s) -High blood presure -Diabities -High cholestrole -Other	**n (%)** 18 (60.0%) 14 (46.7%) 19 (63.3%) 11 (36.7%)	**n (%)** 16 (53.3%) 10 (33.3%) 12 (40.0%) 14 (46.7%)	χ² (1) = 0.271 χ² (1) = 1.111 χ² (1) = 3.270 χ² (1) = 0.617	0.602 0.292 0.071 0.432
Non-sedative Medication Use -Yes -No	**n (%)** 25 (83.3%) 5 (16.7%)	**n (%)** 23 (76.7%) 7 (23.3%)	χ² (1) = 0.417	0.519
Sedative Medication Use -Yes -No	**n (%)** 7 (23.3%) 23 (76.7%)	**n (%)** 7 (23.3%) 23 (76.7%)	χ² (1) = 0.000	1.000

^a^ Measured by Abbreviated Mental Test Score, range 0–10

### Main outcomes: sleep quality and fatigue

Statistical analysis of pre- and post-intervention scores after the 30-day trial period shows that a statistically significant differences were found in both sleep quality and fatigue levels in the intervention group as shown in Table 2. Mean PSQI score in the intervention group decreased from 11.5 ± 2.8 to 9.2 ± 3.1(Mann–Whitney U = 208.5, z = -3.621, *P* < 0.001), indicating improved sleep quality likely associated with the intervention. Consequently, the mean IFS score, decreased from 35.1 ± 3.5 to 32.4 ± 4.9 (t (58) = 2.816, *P* = 0.007), suggesting a statistically significant reduction in perceived fatigue related to the mentioned intervention. On the other hand, small improvements were observed in the PSQI and IFS scores of the control group, but no statistically significant changes were observed between pre- and post-intervention scores for either outcome. Mean PSQI score in the control group decreased from 11.0 ± 3.1 to 10.9 ± 3.2 (t (29) = 0.191, *P* = 0.850), indicating no statistically significant improvement in sleep quality associated with the standard care. Similarly, the mean IFS score, decreased from 34.5 ± 3.5 to 33.9 ± 4.0 (Z = -1.006, *P* = 0.314), suggesting no statistically significant improvement on perceived fatigue related to the mentioned standard care. The between-group differences in post-intervention scores, after adjustment for baseline values, indicate a significant differential change over time between the two groups.

**Table 2:** Main findings ^a^

Variable	Intervention Group (*n* = 30)	Control Group (*n* = 30)	Statistic	*P* value
Sleep quality (PSQI) ^b^ -Before -After -Difference -Within-group test	**mean ± (SD)** 11.5 ± (2.8) 9.2 ± (3.1) 2.3 ± (2.6) t (29) = 4.723 | *P* < 0.001	**mean ± (SD)** 11.0 ± (3.1) 10.9 ± (3.2) 0.03 ± (1.8) t (29) = 0.191 | *P* = 0.850	t (58) = 0.644 t (58) = -2.119 U = 208.5 (z = -3.621)	0.522 0.038 < 0.001
Fatigue (IFS) ^c^ -Before -After -Difference -Within-group test	**mean ± (SD)** 35.1 ± (3.5) 32.4 ± (4.9) 2.7 ± (2.9) z = -3.835 | *P* < 0.001	**mean ± (SD)** 34.5 ± (3.5) 33.9 ± (4.0) 0.6 ± (2.7) z = -1.006 | *P* = 0.314	U = 429.5 (z = -0.305) U = 337.0 (z = -1.676) t (58) = 2.816	0.761 0.094 0.007
Decrease in Sleep onset latency (min) ^d^	**mean ± (SD)** 10.0 ± (9.0)	**mean ± (SD)** 2.0 ± (6.5)	U = 244.0 (z = -3.537)	< 0.001
Increase in over-night sleep duration (hours) ^e^	**mean ± (SD)** 0.2 ± (0.7)	**mean ± (SD)** 0.07 ± (0.6)	U = 400.0 (z = -0.862)	0.389

^a^ In between groups differences, depending on the result of normality tests, for continuous variables independent samples t-tests or Mann–Whitney U tests were used, Chi-square and exact Chi-square tests were applied for categorical variables. When necessary, Wilcoxon signed-rank or paired t-tests were used to examine within-group changes

^b^ Measured by Pittsburgh Sleep Quality Index, range 0–21

^c^ Measured by Iowa Fatigue Scale, range 11–55

^d^ Measured by Pittsburgh Sleep Quality Index, range 0–60+ (min)

^e^ Measured by Pittsburgh Sleep Quality Index, range 0–5+ (hour)

### Secondary findings: sleep onset latency, total sleep time and effect of age

Also shown in Table 2, analyzing the data collected from PSQI questionnaire and participant personal logs shows that the intervention group’s sleep onset latency (i.e. the amount of time it takes to fall asleep) had significantly decreased (mean reduction of 10.0 ± 9.0 min compared to 2.0 ± 6.5 min in control group, Mann–Whitney U = 244.0, z = -3.537, *P* < 0.001), while no significant change was found in the control group. However, despite the total nighttime sleep duration increasing in the intervention group (mean increase of 0.2 ± 0.7 h ~ 12 min compared to 0.07 ± 0.6 h ~ 4 min in control group), these changes were not statistically significant (Mann–Whitney U = 400.0, z = -0.862, *P* = 0.389). our secondary analysis, As shown in Table 3, shows that a significant main effect of age can be observed, suggesting that fatigue level differed across age groups (F (2,2) = 38.453, *P* = 0.025). However, the interaction between group and age was not statistically significant (F (2,54) = 0.023, *P* = 0.977), indicating that the effect of the intervention on fatigue did not differ by the age group.

**Table 3:** Effects of group and age on changes in fatigue scores (IFS)

Effect^a^	df 1	df 2	F value	*P* value
Group (intervention vs. control)	1	18.057	118.406	< 0.001
Age group	2	2	38.453	0.025
Group × age interaction	2	54	0.023	0.977
Overall model	1	2.244	20.227	0.037

^a^ Two-way analysis of variance (ANOVA)

### Descriptive post-intervention participant feedback

Based on the Feedback gathered by post-intervention interview, 24 out of 30 people in the intervention group described their experience of using the provided white noise to be relaxing, even in those for whom the use of white noise had little or no effect on their sleep quality. Four out of these 24 people, mentioned that listening to white noise has led them to their distant fond memories and it helped them with falling asleep. Four people out of 30 did not enjoy using white noise and described using it to be disrupting to their process of falling asleep. Two of these four people’s nighttime sleep routine was praying before falling asleep, and they said that having these sounds playing in the background was disturbing for them. Two out of 30 people in the intervention group said that listening to white noise was neither relaxing nor disturbing for them. 10 out of 30 people in the intervention group said that listening to white noise had helped them with masking the disturbing environmental sounds that had distorted their ability to fall asleep before. No changes to the number of mid-sleep awakenings have been reported. No additional analysis was conducted on the descriptive data that was gathered in these interviews.

### Adverse effects

No adverse effects or safety concerns were reported by participants in either group.

## Discussion

This RCT was conducted to examine the effects of white noise on sleep quality and fatigue in older adults.

Our results show that using white noise in addition to standard care, was associated with statistically significant improvements in sleep quality and reduction in fatigue among community-dwelling older adults. These findings are consistent with prior similar studies that reported on the effects of white noise and its ability to mask environmental noise as well as reducing sleep onset latency [22, 26, 28].

Results from previous studies, also align with our findings in terms of the effect of white noise on improving sleep quality.

Research done by Farokhnezhad et al. (2016) and Oshvandi et al. (2020), showed that white noise was associated with improved sleep comfort and reduced perceived impact of environmental disturbances in hospitalized older adults [26, 28]. Our findings extend this evidence by showing the effectiveness of white noise in a home setting and its associations with improved sleep and reduced fatigue.

Furthermore, participants in our study reported decreased sleep onset latency, a result similar to the findings from Yin et al. (2024) and Ebben et al. (2021), where white noise was shown to be effective in reducing time to fall asleep and improving subjective sleep quality [19, 23].

Although the total nighttime sleep duration increased slightly in the intervention group, this change was not statistically significant, suggesting that improvements in perceived sleep quality and fatigue measured by PSQI and IFS scales, might be more related to decreased sleep onset latency and reduced sleep disruptions than to increased sleep duration [19, 36].

Based on our findings from previous studies, there are several hypotheses that may help explain the observed association between improved sleep quality and lower fatigue levels, such as: 1) Metabolic regeneration and elimination of cellular waste: during sleep, the brain removes metabolic waste and oxygen reactions more quickly than when awake; anabolic hormones (such as growth hormone) are also secreted, which help regenerate tissues and reduce the feeling of fatigue. This theory refers to the mechanism of the “glymphatic system” [40]. 2) Energy restoration and improvement of neurotransmitter activity: Adequate sleep enhances the activity of the neurotransmitters dopamine and norepinephrine, which can lead to reduced fatigue and improved mental and physical performance [41, 42]. 3) Enhanced wakefulness mechanism” Insufficient or poor-quality sleep weakens the wakefulness system and reduces the energy required for alertness and activity. With improved sleep, this system is restored and provides sufficient energy for daily functioning [43].

Based on our findings, improvements in perceived fatigue appear to be associated with concurrent improvements in sleep quality, as reflected by PSQI scores These findings support the mentioned hypothesis that better sleep quality leads to lower perceived fatigue levels. Thus, improvements in fatigue may be interpreted as secondary to improvements in sleep quality, not as a direct physiological effect of white noise.

While no prior RCTs directly investigated the impact of white noise on fatigue in older adults, our findings are in line with studies by Genç et al. (2020) and Hawker et al. (2010), which highlighted the association between improved sleep and reduced fatigue [36, 44].

It is notable to say that the finding of Table 3 suggests that while fatigue levels differed significantly depending on how old the participants were, the beneficial effect of white noise on fatigue was consistent across age groups. Qualitative feedback gathered from post-intervention interviews supported the quantitative findings as well. However, dispite the fact that several of our participants described that using white noise was distracting or incompatible with their pre-sleep routines, such as prayer or complete silence, the majority of them described it to be calming and relaxing.

Participants with conditions such as sleep apnea, restless leg syndrome or nightmares, despite describing the use of these sounds as relaxing, did not show significant changes in their duration of sleep or sleep onset latency.

However, not all evidence points to the effectiveness of white noise. A systematic review by Ridey et al. (2021), noted that a review of 38 studies found that the evidence for the effectiveness of white noise was very weak. Some studies even reported that white noise may cause further sleep disruption [20]. These findings are not consistent with the findings of our study; however, it should be noted that the studies included in this systematic review were very diverse in terms of sound type, target population, and length of the intervention. These heterogeneities could be the reasons for the differences in results with the present study. Consequently, comparisons of the results of our study with this systematic review should be made with caution.

Another finding in our study, was the individual’s preference for using the sounds that were provided to them. According to our findings, the order of most preferred sounds was river (36.7%), forest (30.0%), rain (26.7%), and sea waves (6.7%). Based on the findings from interviews, it seems that individual taste and preference in choosing the type of sound can affect the effectiveness of the intervention. These findings can depend on factors such as previous experiences, cultural background, level of mental familiarity with the sound and even the mental health of individuals.

Overall, our findings suggest that white noise can be used as a simple, safe, cost-effective, non-pharmacological strategy to improve sleep and reduce fatigue in older adults, particularly those living in noisy environments.

### Strengths, limitations and future research

The randomized controlled design of this study is one of its strengths. Moreover, by focusing on community-dwelling older adults and letting the participants be involved in administering the intervention in their own homes, this study offers insight into the practical use of white noise in real-life environments. By including multiple health centers and a relatively diverse participant pool, we enhance the internal validity of the study. In addition, the combination of reliable quantitative tools with post-intervention participant feedback provided an overall understanding of participants’ experiences and responses to the intervention. Despite these strengths, we should acknowledge several limitations as well. First, the study sample was drawn from a specific geographic region in the city of Mashhad, which although it may be diverse, it can limit the generalizability of the results to other regions or populations with different cultural or environmental factors. Second, the use of self-reported questionnaires may introduce subjectivity and potential recall bias. Third, even though the intervention duration was significantly longer than those in similar prior studies, it was limited to 30 days due to limitations of self-reporting data gathering tools. Additionally, due to the design of the study that aimed to understand the effect of white noise on community-dwelling individuals, our study could not control all possible confounding variables including background environmental noise. Although this may have introduced variability in noise exposure, we think it enhances the ecological validity of the findings, and it is more reflective of typical sleep environments of community-dwelling older adults. Lastly, similar to other sleep intervention studies, sedative medication use represents a potential confounding factor. In the present trial, the proportion of sedative use was similar between the intervention and control groups, reducing the likelihood of systematic bias. However, interpretation of the observed improvements in sleep quality and fatigue should be made with caution and considered in the context of routine medication use among older adults. Future research could compare white noise with other non-pharmacological interventions such as lavender aromatherapy, pink noise, nature vs. man-made sounds. Additionally, future studies could explore other settings especially nursing homes as well as, examining multi-dimensional sleep improvement strategies, combining environmental, behavioral, and psychological approaches; and also investigate white noise’s effects on stress and anxiety in older adults. Additionally, future studies with larger sample sizes and multiple follow-up assessments could employ longitudinal analytical approaches, such as generalized estimating equations (GEE) or mixed-effects models, to more comprehensively account for within-subject correlations and evaluate changes in outcomes over time.

### Conclusions

Findings from our RCT suggest that white noise can promote relaxation and shorten sleep onset latency. As a result, it could be used to improve overall sleep quality and may be associated with reductions in fatigue in community-dwelling older adults. Additionally, although our study did not directly measure the underlying mechanisms of white noise’s calming effects or its impact on environmental sounds, participants’ experiences, together with findings from previous studies, suggest that white noise may help reduce perceived environmental distractions and exert calming effects in older adults. Healthcare providers and caregivers may consider white noise as part of a comprehensive routine sleep hygiene approach for older adults due to the intervention’s potential benefits, ease of use, and safety.

## Supplementary Information

Supplementary Material 1.

Supplementary Material 2.

Supplementary Material 3.

Supplementary Material 4.

## Data Availability

The datasets generated and/or analyzed during the current study are not publicly available as the participant consent forms did not address open public access to the data and due to ethical approval limitations by the Ethics Board at the Mashhad University of Medical Sciences (MUMS). Data are available upon request from the corresponding author on reasonable request and subject to Ethics Board review.
