# Supplementary material for: The effect of white noise on sleep quality and fatigue in community-dwelling older adults: a randomized controlled trial
Source: BMC Geriatr. 2026 May 2;26:883. doi: 10.1186/s12877-026-07311-2 (PMC13321728; doi:10.1186/s12877-026-07311-2)
Supplement: Supplementary file 1 — Supplementary Material 1. [file 12877_2026_7311_MOESM1_ESM.pdf]

Gmail

Amirhossein Vahabzadeh <amirvm1998@gmail.com>

Request for Permission to Use IFS Questionnaire

2 messages

Amirhossein Vahabzadeh <amirvm1998@gmail.com> Fri, Oct 10, 2025 at 9:40 PM
To: hartzarthur@gmail.com

Dear Mr. Hartz,

I hope this message finds you well.
My name is Seyed Amirhossein Vahabzadeh Mousavi, and I am a MSc student at the University of Mashhad Medical Sciences and we are conducting a postgraduate thesis project in Iran titled “The Effect of White Noise on Sleep Quality and Fatigue in Community-Dwelling Older Adults”.

I am writing this email to request your kind permission to use the Iowa Fatigue Scale (IFS) in our research project.

The study aims to “examine the effects of white noise on sleep quality among older adults”. We believe your instrument would be highly suitable and valuable for identifying and examining the fatigued individuals in our project to achieve the study objectives.

Due to financial constraints and lack of institutional funding we kindly request that you waiver the charges that may apply.

Thank you very much for your time and consideration. I greatly appreciate your contribution to advancing research in this area.

Kind regards,
Seyed Amirhossein Vahabzadeh Mousavi
MSc Student in geriatric nursing, Department of Nursing
Mashhad University of Medical Sciences (MUMS)
WhatsApp: +989159167264
amirvm1998@gmail.com

hartzarthur@gmail.com <hartzarthur@gmail.com> Fri, Oct 10, 2025 at 10:02 PM
To: Amirhossein Vahabzadeh <amirvm1998@gmail.com>

Certainly, you may use this instrument for your research. Although it is not a requirement for use, I would appreciate your sending me the abstract of the results of your study.

Arthur Hartz, MD, PhD

[Quoted text hidden]
