# Supplementary material for: The effect of white noise on sleep quality and fatigue in community-dwelling older adults: a randomized controlled trial
Source: BMC Geriatr. 2026 May 2;26:883. doi: 10.1186/s12877-026-07311-2 (PMC13321728; doi:10.1186/s12877-026-07311-2)
Supplement: Supplementary file 2 — Supplementary Material 2. [file 12877_2026_7311_MOESM2_ESM.pdf]

Gmail

Amirhossein Vahabzadeh <amirvm1998@gmail.com>

Permission to use the PSQI - Sleep Measures Request Form (PSQI, etc.) - 2023 - v3

1 message

University of Pittsburgh Center for Sleep and Circadian Science <noreply@qualtrics-research.com> Fri, Oct 10, 2025 at 8:24 PM
Reply-To: University of Pittsburgh Center for Sleep and Circadian Science <trigger@qemailserver.com>
To: amirvm1998@gmail.com

Dear Seyed Amirhossein,

Thank you for your interest in the PSQI. You have permission to use the PSQI in the non-commercially funded research or educational project described in your request. This permission extends only to the specific project described in your request. You will need to submit a separate request if you would like to use the PSQI in other projects. Permission to use the PSQI has the following provisions:

1. Electronic format: If you plan to administer the PSQI in electronic format, you must ensure that the questions and responses are faithfully reproduced from the original form. We do not have the PSQI available in an electronic format for distribution. Do not distribute the PSQI in electronic format to any other individuals and groups outside of your specific request.

2. Translations are distributed through an agreement with MAPI Research Trust. **You must present this permission letter to obtain a translation from MAPI.** The website (https://eprovide.mapi-trust.org/) will indicate what languages they have on file. You will need to work with them to obtain any necessary translations. They will collect the proper user agreement. We do not know if they have the required translation on file. You will need to contact MAPI for that information. If the translations you need are unavailable, MAPI can provide them for a cost. You will need to contact them for the exact price. If you have trouble obtaining the translations, let us know.

If you translate the PSQI yourself, translations must use established linguistic validation methods including (but not limited to) cognitive interviews, translation, and back-translation by two bi-lingual speakers.

All translations remain property of the University of Pittsburgh. They are considered derivative works of the original work.

COPYRIGHT NOTICE FOR TRANSLATION: LICENSEE shall ensure that the following copyright notice is placed on all use of the translations of the PSQI: © 1989 and 2010, University of Pittsburgh. All rights reserved. Translation [insert year], by _______________.

Once translation is completed, please send a copy to IILA@pitt.edu and vlachosc@upmc.edu.

Additionally, please send a copy of the translation to our distribution partners Mapi Research Trust by submitting a request through their ePROVIDE™ platform.

1. **Go to Submit a request**
2. **If you haven't registered yet, you'll be asked to sign up for free**
3. **Complete the request form and attach a copy of the translation.**

3. Referencing: All publications, presentations, reports, or developments resulting from or relative to the use of this material must be referenced as follows:
The Pittsburgh Sleep Quality Index: A New Instrument for Psychiatric Practice and Research (Authors Daniel J. Buysse, Charles F. Reynolds III, Timothy H. Monk, Susan R. Berman , and David J Kupfer, © University of Pittsburgh 1989)

4. Limitation of Liability: The University of Pittsburgh disclaims any and all liability, including but not limited to consequential, incidental, direct, indirect, special, punitive, or other damages whatsoever arising out of the use of or inability to use the PSQI.

General information regarding the PSQI, including the instrument, original article, and scoring information, is available at our website, https://sleep.pitt.edu/psqi.

Note that Question 10 is not used in scoring the PSQI. This question is for informational purposes only, and may be omitted during data collection per requirements of the particular study.

Good luck with your research!

Sincerely,

Daniel J. Buysse, MD
Distinguished Professor of Psychiatry, Medicine,
and Clinical and Translational Science
UPMC Endowed Chair in Sleep Medicine
University of Pittsburgh School of Medicine
3811 O'Hara St., E-1123
Pittsburgh, PA 15213

This email may contain confidential information of the sending organization. Any unauthorized or improper disclosure, copying, distribution, or use of the contents of this email and attached document(s) is prohibited. The information contained in this email and attached document(s) is intended only for the personal and confidential use of the recipient(s) named above. If you have received this communication in error, please notify the sender immediately by email and delete the original email and attached document(s).

Recipient Data:
Time Finished: 2025-10-10 12:54:33 EDT
IP: 162.19.204.60
ResponseID: R_2RPUjoTeH9XNa4K
Link to View Results: Click Here
URL to View Results: https://pitt.iad1.qualtrics.com/apps/single-response-reports/reports/qqaN6Dxq96-n73MlGGoTMPk9zPc-v933DSrilDvQl9mJ6QKAoDqghqFMD1sOvcmZDHwL0K0rMtCCWOqHz1KnZisaWfqx1sWmTtsmjv29RTFjkbZkqRLRJZniQa-ZXLIBzqZyif97Dut8za9XlG7dMBToJvqwfcn8wTrOTlyG58mTAy3ucSWTddGtD0FnN2AtcB71ygmzu1Kh7_7Ix161gjrfmUorCoGh48VzjrE4z8FXToM-TsDOufTjzwsMOOao4-QG3LxLRjLQ13mlC8HzFybDkHCDjbyDeYRZKQZgYPHBbDVcb6OO13QWyw507cKZbLyfMZER0DJ6s5kz1IrOEg

---

Response Summary:

Name
First Name Seyed Amirhossein
Last Name Vahhabzadeh Mousavi

Credentials/Degree
MSc geriatric nursing

Email address
amirvm1998@gmail.com

Instrument Requested (please submit a separate request for each):
Pittsburgh Sleep Quality Index (PSQI)

You are requesting this instrument in your role as a...
Student or trainee

Please answer the following regarding how you will use the instrument:
Unfunded research project (school, college, or university project)

Company, Institution or Organization
Mashhad University of medical sciences

Country
Other Country (Please indicate): -- Iran

Study Title and Brief Description of Project (please be complete)
title: The Effect of White Noise on Sleep Quality and Fatigue in Community-Dwelling Older Adults
this research is a postgraduate thesis project.

Modification requested (If none, leave blank):
