# Supplementary figures and images for: The effect of white noise on sleep quality and fatigue in community-dwelling older adults: a randomized controlled trial
Source: BMC Geriatr. 2026 May 2;26:883. doi: 10.1186/s12877-026-07311-2 (PMC13321728; doi:10.1186/s12877-026-07311-2)

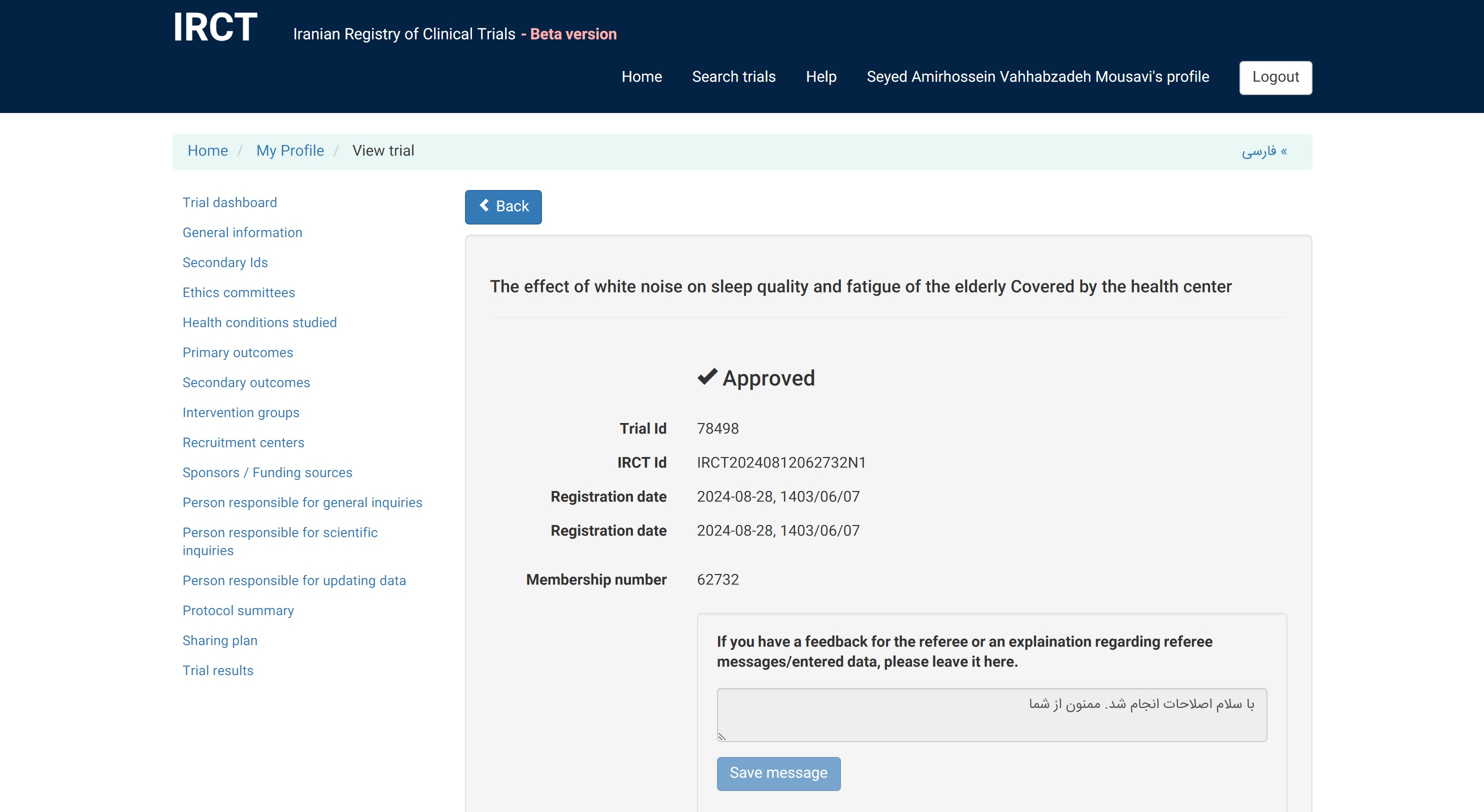

Supplement: Supplementary file 3 — Supplementary Material 3. [file 12877_2026_7311_MOESM3_ESM.jpg]
